# Lentiviral Vector-Mediated Correction of a Mouse Model of Leukocyte Adhesion Deficiency Type I

**DOI:** 10.1089/hum.2016.016

**Published:** 2016-09-01

**Authors:** Diego Leon-Rico, Montserrat Aldea, Raquel Sanchez-Baltasar, Cristina Mesa-Nuñez, Julien Record, Siobhan O. Burns, Giorgia Santilli, Adrian J. Thrasher, Juan A. Bueren, Elena Almarza

**Affiliations:** ^1^ Division of Hematopoietic Innovative Therapies, Centro de Investigaciones Energéticas, Medioambientales y Tecnológicas (CIEMAT), and Centro de Investigación Biomédica en Red de Enfermedades Raras (CIBERER), Madrid, Spain.; ^2^ Instituto de Investigación Sanitaria Fundación Jiménez Díaz (IIS-FJD, UAM), Madrid, Spain.; ^3^ Section of Molecular and Cellular Immunology, University College London Institute of Child Health, London, United Kingdom.; ^4^ Department of Immunology, Royal Free London NHS Foundation Trust, London, United Kingdom.; ^5^ University College London Institute of Immunity and Transplantation, London, United Kingdom.; ^6^ Great Ormond Street Hospital Foundation Trust NHS Trust, London, United Kingdom.

## Abstract

Leukocyte adhesion deficiency type I (LAD-I) is a primary immunodeficiency caused by mutations in the *ITGB2* gene and is characterized by recurrent and life-threatening bacterial infections. These mutations lead to defective or absent expression of β_2_ integrins on the leukocyte surface, compromising adhesion and extravasation at sites of infection. Three different lentiviral vectors (LVs) conferring ubiquitous or preferential expression of CD18 in myeloid cells were constructed and tested in human and mouse LAD-I cells. All three hCD18-LVs restored CD18 and CD11a membrane expression in LAD-I patient-derived lymphoblastoid cells. Corrected cells recovered the ability to aggregate and bind to sICAM-1 after stimulation. All vectors induced stable hCD18 expression in hematopoietic cells from mice with a hypomorphic *Itgb2* mutation (CD18^HYP^), both *in vitro* and *in vivo* after transplantation of corrected cells into primary and secondary CD18^HYP^ recipients. hCD18^+^ hematopoietic cells from transplanted CD18^HYP^ mice also showed restoration of mCD11a surface co-expression. The analysis of *in vivo* neutrophil migration in CD18^HYP^ mice subjected to two different inflammation models demonstrated that the LV-mediated gene therapy completely restored neutrophil extravasation in response to inflammatory stimuli. Finally, these vectors were able to correct the phenotype of human myeloid cells derived from CD34^+^ progenitors defective in ITGB2 expression. These results support for the first time the use of hCD18-LVs for the treatment of LAD-I patients in clinical trials.

## Introduction

Leukocyte adhesion deficiency type I (LAD-I) is an autosomal recessive primary immunodeficiency caused by deficient cell surface expression of β_2_ integrins. As a consequence, neutrophils fail to firmly adhere to the inflamed endothelium and to extravasate from blood to infection sites. The molecular basis underlying LAD-I are mutations in the *ITGB2* gene that encodes for the β_2_ common integrin subunit (CD18).^1–3^

LAD-I patients suffer from recurrent and life-threatening infections that appear early in childhood.^4^ Two different phenotypes of LAD-I have been described^5^: a severe phenotype, when levels of CD18 expression are lower than 2% of normal level, and a moderate phenotype, when levels of CD18 expression are 2–30% of the normal level. Hematopoietic stem cell transplantation (HSCT) is currently the only curative treatment for LAD-I.^6^

After several *in vitro* studies,^7–10^ the first attempt to treat LAD-I by gene therapy (GT) was carried out in 2000 when two patients were enrolled in a phase-I GT clinical trial.^11,12^ Mobilized CD34^+^ HSCs were collected from peripheral blood (PB), transduced with a GALV-pseudotyped γ-RV, and infused back into the patients without any conditioning. A small percentage of corrected myeloid cells (up to 0.04%) were detected in PB up to 4 weeks after transplantation, but no corrected cells were detected 2 months after transplantation.

Apart from the clinical studies shown above, additional preclinical GT studies have been carried out in a canine leukocyte adhesion deficiency (CLAD) model typical of Irish setter dogs.^13,14^ The therapeutic efficacy of different vectors has been evaluated in this model using nonmyeloablative conditioning. Independently of the vector—γ-RV, foamy viral vector (FV), or self-inactivating lentiviral vector (SIN LV)—several animals were rescued from the disease when the expression of the canine CD18 (cCD18) cDNA was driven by the murine stem cell virus (MSCV) LTR promoter/enhancer.^15,16^ In a new attempt to improve the safety of this GT approach, either FV or SIN LVs carrying weaker promoters were designed. However, in these cases, the outcome of treated dogs was less conclusive.^16–18^ The lack of a full myeloablative conditioning and the transduction of target cells at low multiplicity of infection (MOI) could account for the modest therapeutic effects observed in these studies.

In our studies lentiviral vectors carrying three different promoters to drive the expression of hCD18 were constructed. The human PGK promoter has been extensively investigated in preclinical studies demonstrating its ubiquitous, moderate, and stable activity *in vivo.*^19,20^ More recently, clinical GT studies have been also conducted showing the efficacy of this promoter in metachromatic leukodystrophy patients.^21^ The A2UCOE promoter, from the human *HNRPA2B1-CBX3* locus, has also been shown to provide efficient therapeutic correction in different preclinical disease mouse models such as SCID-X1^22^ and recombination activating gene 2 (*RAG2*)-SCID,^23^ with no significant adverse effects, and with little or no methylation.^24–26^ We have also used a chimeric promoter that is a fusion of the minimal 5′-flanking regions *of cFES* and the *CTSG* genes, which encode for proteins expressed during neutrophil maturation. This promoter drives preferential expression in myeloid cells and has been successfully used for hematopoietic *ex vivo* GT in a mouse model of X-CGD^27^ and is currently in clinical trial.

To evaluate the therapeutic efficacy of these vectors, a mouse model of LAD-I with a hypomorphic mutation in *CD18* (CD18^HYP^)^28^ was used. Homozygous mutant CD18^HYP^ mice exhibit impaired inflammatory responses, mild leukocytosis, hyperplasia in spleen and BM, and increased content of hematopoietic progenitors and HSCs in their BM, compared with wild-type (WT) mice.^29^ Additionally, *in vitro* studies with human LAD-I lymphoblastic cells (LCs) and with cord blood CD34+ cells with a downregulated expression in CD18 were performed. Our data strongly show the therapeutic value of hCD18-LVs, suggesting that these vectors would efficiently restore the clinical signs of LAD-I patients.

## Materials and Methods

### *Ex vivo* gene therapy

For *ex vivo* GT experiments, overnight-transduced lin^−^ cells were collected and washed and 3–5 × 10^5^ cells were intravenously administered into lethally irradiated female CD18^HYP^ mice. PB was monthly collected and analyzed for the expression of hCD18 and the different murine CD11 subunits. Genomic DNA (gDNA) was used for vector copy number (VCN) determination. Three months after transplantation, animals were analyzed for hCD18 expression in the different leukocyte subpopulations. Transplanted mice were culled at 4 months posttransplantation (mpt) and total bone marrow cells (BMCs) were analyzed. In total, 3 × 10^6^ BMCs were transplanted mouse to mouse into myeloablated secondary recipients that were followed up for 9 months.

### Air-pouch inflammation model

The air pouch (AP) was generated by dorsal subcutaneous injection of air on day 0 under isoflurane anesthesia. On day 3 the pouches were re-inflated and, on day 5, 40 ng of mouse recombinant TNF-α in phosphate buffered saline (PBS) with 0.5% carboxymethylcellulose (CMC) as inert carrier was injected into the matured pouches. Four hours after TNF-α administration, mice were sacrificed by CO_2_ inhalation, and PB was collected by heart puncture and the total number of neutrophils determined. The APs were then flushed with, and the total neutrophils number in the AP was also determined. Emigration ratio for total neutrophils was calculated as follows: Emigration Ratio = (AP neutrophils/PB neutrophils) × 100. In the case of GT-treated animals, hCD18^+^ or GFP^+^ neutrophils were determined in PB and in the AP, and emigration ratio was calculated as follows: Emigration Ratio = (hCD18^+^ or GFP^+^ AP neutrophils/hCD18^+^ or GFP^+^ PB neutrophils) × 100

### Lipopolysaccharide-induced asthma model

Mice were intranasally administered with 50 μl of 0.3 μg/μl lipopolysaccharide (LPS, from *Escherichia coli* 0111:B4; Sigma-Aldrich, St. Louis, MO) or PBS under isoflurane anesthesia. Twenty-four hours after LPS/PBS administration, bronchoalveolar lavages (BALs) were performed. Mice were euthanized immediately before lavage by lethal injection of avertin (synthetized in the laboratory from 2,2,2-tribromoethanol and 2-methyl-2-butanol, both from Sigma-Aldrich) and exsanguination. Then a 18G blunt fill needle (BD, 305180) was inserted through a small incision in the trachea and lavages were collected. Cell number analysis and FACS analysis (Ly6G/CD11c) were performed in those samples. Absolute numbers of neutrophils were determined as follows: Total Cell Number × (% Ly6G^High^ CD11c^−^ cells). Absolute numbers of migrating neutrophils were normalized to the values obtained in the CD18^HYP^ animals.

## Results

### Lentiviral-mediated phenotypic correction of LAD-I lymphoblast cell lines

**Figure 1.**
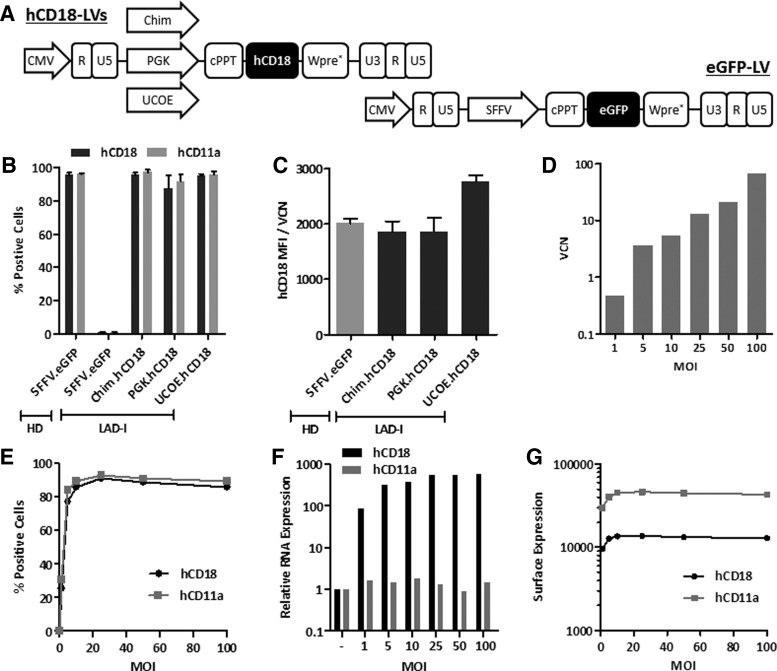
Transduction of LAD-I lymphoblastic cells and endogenous regulation of hCD18 membrane expression. **(A)** hCD18 and eGFP-LV constructs used in the different experiments. **(B–G)** LAD-I and HD LCs were transduced with hCD18-LVs and control eGFP-LVs. **(B)** Percentage of hCD18^+^ and hCD11a^+^ cells 5 days after transduction at an MOI of 10 i.u./cell. **(C)** Relative hCD18 surface expression corrected with the VCN. **(D)** LAD-I LCs VCN transduced at increasing MOIs (from 1 to 100 i.u./cell) with LV:Chim.hCD18. **(E)** Percentage of hCD18/hCD11a-expressing cells after transduction with increasing MOIs with LV:Chim.hCD18. **(F)** hCD18 and hCD11a mRNA expression levels relative to untransduced LCs. **(G)** hCD18 and hCD11a surface expression levels. HD, healthy donor; LAD-I, leukocyte adhesion deficiency type I; LC, lymphoblastic cell; LV, lentiviral vector; MOI, multiplicity of infection; VCN, vector copy number.

We generated three different SIN-LVs in which the expression of hCD18 was driven by two ubiquitous promoters, PGK and UCOE promoters,^19–22^ and by the chimeric promoter, which has preferential activity in mature myeloid cells.^27^ As a negative control a SFFV-eGFP-LV was used in all experiments (Fig. 1A and Supplementary Methods; Supplementary Data are available online at www.liebertpub.com/hum).

In a first set of experiments we investigated the efficacy of the hCD18-LVs vectors to confer hCD18 expression in human LAD-I LCs, with no detectable expression of hCD18 (Fig. 1B). After transduction of these cells with any of the hCD18-LVs, almost 100% of the cells expressed hCD18 on the cell membrane (Fig. 1B). Moreover, since the translocation of hCD11a to the cell membrane is dependent on CD18 expression, the transduction of LAD-I cells with any of the hCD18-LVs efficiently restored the membrane expression of hCD11a (Fig. 1B).

To determine hCD18 expression levels conferred by the different LVs in LAD-I LCs, mean fluorescence intensity (MFI) values as well as mean copy numbers of each LV were determined after transduction in each population, and MFI values were normalized per copy of integrated provirus (MFI/VCN) (Fig. 1C). Although the UCOE promoter seemed to confer the highest membrane expression levels of hCD18, no significant differences were noted between LAD-I cells transduced with any of the hCD18-LVs, either when compared among themselves, or when compared with LCs from a healthy donor (HD) (*p* > 0.05). This indicates that the three LV constructs were able to confer physiological levels of hCD18 in human LAD-I LCs.

Because CD18 is expressed in the cell membrane in association with CD11, we speculated that the ectopic membrane expression of hCD18 might be regulated by endogenous physiological levels of hCD11. To validate this hypothesis, LAD-I LCs were transduced with the Chim.hCD18-LV at increasing MOIs. As expected, increases in the MOI mediated a progressive increase in the VCN/cell (Fig. 1D), with most cells being positive for hCD18 expression at MOIs of 10 i.u./cell or higher (Fig. 1E). Also, hCD18 mRNA levels—but not hCD11a mRNA levels—progressively increased in parallel to VCN/cell increases (Fig. 1F). However, the ectopic expression of both CD18 and CD11a in the membrane of LAD-I-transduced cells reached a plateau at an MOI of 10 i.u./cell (Fig. 1G).

**Figure 2.**
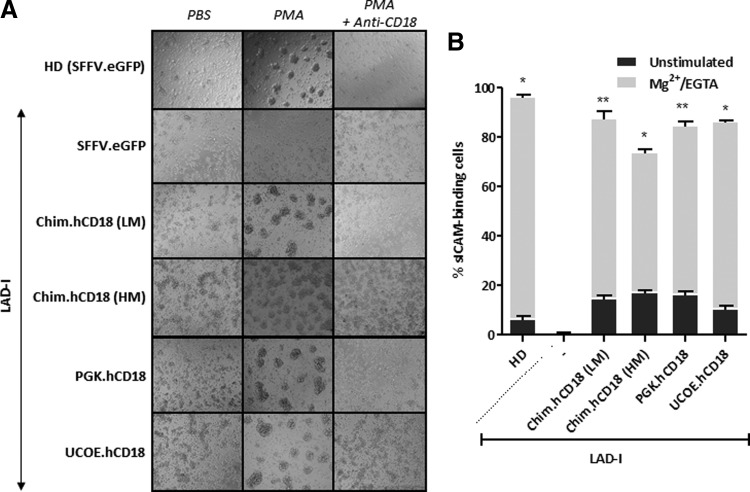
Phenotypic correction of LAD-I lymphoblastoid cells. LAD-I and HD LCs were transduced with hCD18-LVs and control eGFP-LVs at an MOI of 10 i.u./cell. **(A)** PMA-induced aggregation assays. Cells were incubated with either PBS or PMA and let to aggregate for 1 hr. Anti-CD18 blocking antibody was also added to demonstrate that the observed aggregation was dependent on hCD18. LV:Chim.hCD18 was used at two different MOIs: low MOI (LM 10 i.u./cell) and high MOI (100 i.u./cell). **(B)** Soluble ICAM-1 binding assay. LFA-1 integrins were activated and thus cells were let to bind to sICAM-1 in the presence or absence of Mg^2+^ and EGTA for Ca^2+^ chelation. Chim LV was used at two different MOIs: low MOI (LM 10 i.u./cell) and high MOI (100 i.u./cell). The significance of differences between groups is expressed as *p < 0.05 and **p < 0.01. PBS, phosphate buffered saline; PMA, 4 beta-phorbol-12-myristate-13-acetate. See Supplementary Table S1 for list of antibodies used.

To investigate whether restoration of expression of hCD18 corrected the function of LAD-I cells, we first performed aggregation assays in LAD-I LCs. While 4 beta-phorbol-12-myristate-13-acetate (PMA) induced the aggregation of eGFP-transduced HD LCs, no aggregation was induced in eGFP-transduced LAD-I LCs (Fig. 2A and Supplementary Methods). Significantly, LAD-I LCs that had been transduced with any of the hCD18-LVs recovered the ability to aggregate after PMA stimulus, indicating that the ectopic expression of hCD18 was functional. The aggregation observed in HD cells and also in corrected LAD-I cells was abrogated in the presence of an anti-hCD18 blocking antibody, confirming that the observed aggregation was dependent on hCD18 expression (Fig. 2A).

In a second functional assay we measured the ability of corrected LCs to bind to a soluble form of the β_2_-integrin ligand ICAM-1 (sICAM-1) after a proper stimulus. Nonactivated HD LCs showed a low basal sICAM-1 binding capacity that markedly increased after activation (Fig. 2B and Supplementary Methods). LAD-I cells did not show any sICAM-1 binding ability, either in the absence or in the presence of activating agents. However, when LAD-I cells were transduced with any of the hCD18 LVs, these cells became able to bind to sICAM-1 at levels comparable to those observed in HD cells (Fig. 2B). Similar outcomes in the PMA-induced aggregation and in the sICAM-1 binding assay were observed in LAD-I LCs transduced with Chim.hCD18 vector at 10 and 100 i.u./cell (Fig. 2A, B).

### *In vitro* and *in vivo* lentiviral-mediated phenotypic correction of CD18^HYP^ mice

To test the efficacy of our LVs in an *in vivo* model we used CD18^HYP^ mice, characterized by reduced levels of mCD18 expression, high numbers of white blood cells (WBCs), and reduced neutrophil emigration capacity upon an inflammation stimulus.^28,29^

**Figure 3.**
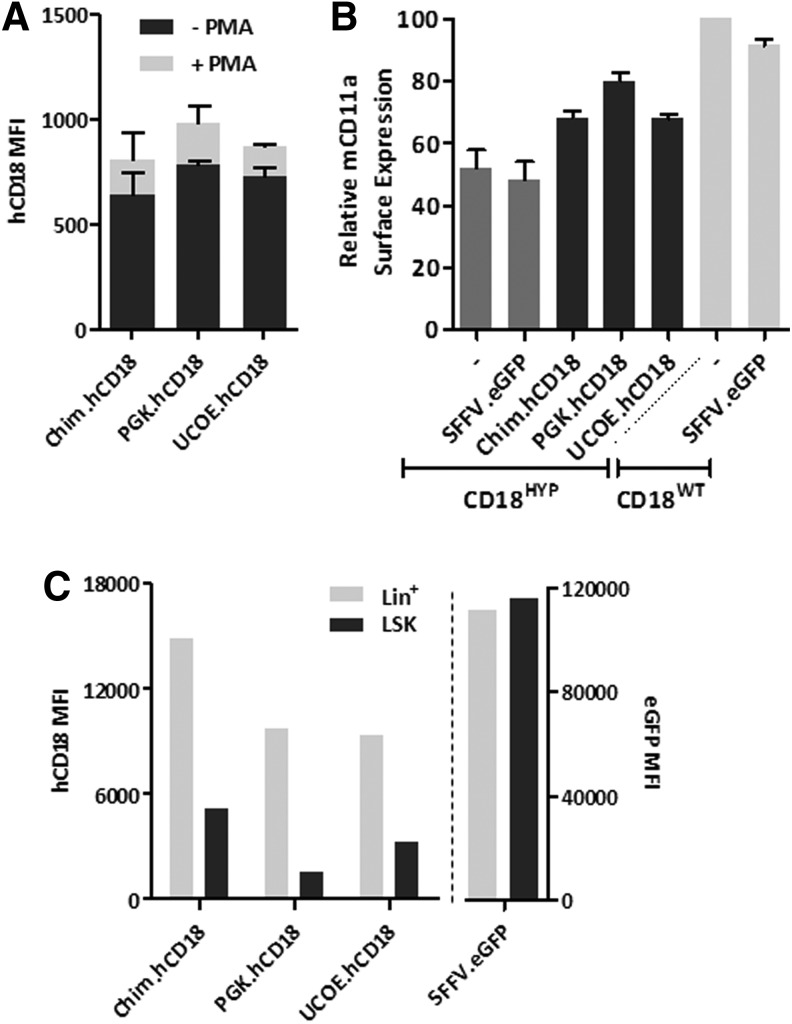
*In vitro* transduction of mouse hematopoietic progenitors. BM lin^−^ cells from CD18^HYP^ mice were transduced for *in vitro* analyses at an MOI of 20 i.u./cell. **(A)** Surface expression of mCD11a in transduced and untransduced myeloid-differentiated CD18^HYP^and CD18^WT^ lin^−^ cells. mCD11a surface expression levels were determined by flow cytometry and then normalized to those found in untransduced CD18^WT^ cells. **(B)** hCD18 regulation in response to PMA in neutrophils generated *in vitro* from transduced CD18^HYP^ lin^−^ cells. hCD18^+^ cells were determined by flow cytometry before and after PMA stimulation**. (C)** hCD18 expression in LSK cells and Lin^+^ BMCs *in vitro.* See Supplementary Table S1 for list of antibodies used.

Lin^−^ BM cells from CD18^HYP^ mice were transduced with the hCD18-LVs and differentiated toward the myeloid lineage *in vitro*. After differentiation 30–40% hCD18^+^ cells were detected, with no significant differences among the different hCD18-LVs (Supplementary Fig. S1A and Supplementary Methods). Similar levels of membrane hCD18 expression were noted in these cells (Supplementary Fig. S1B), and in all instances PMA stimulation increased the expression level of hCD18 (Fig. 3A), mimicking the physiological response of endogenous CD18 to stimulation.^10^ When the expression of the endogenous CD11a was investigated, we observed that either untransduced or eGFP-transduced CD18^HYP^ cells expressed about 50% of the levels corresponding to WT cells. These levels increased to about 70–80% of WT levels after transduction with any of the hCD18-LVs (Fig. 3B), showing that exogenous hCD18 is able to bind to the endogenous mCD11a subunit, forming a β_2_ chimeric integrin.

To investigate the membrane expression levels conferred by the different hCD18 LV in hematopoietic stem and progenitor cells (HSPCs), hCD18 MFI values were determined in undifferentiated and myeloid differentiated CD18^HYP^ Lin^−^Sca1^+^cKit^+^ (LSK) BM cells previously transduced with the different hCD18-LVs. As expected, when these cells were transduced with an eGFP-LV, no differences in eGFP expression were observed between the different populations (Fig. 3C and Supplementary Table S1 for list of antibodies used). Transduction with hCD18-LVs conferred hCD18 expression levels that were much lower, although detectable, in LSK cells compared with levels determined in the more differentiated cells (Fig. 3C), indicating that all LVs mimicked the physiological expression of CD18 in the different hematopoietic compartments.^30^

When clonogenic assays with transduced samples were performed, similar numbers of colonies were generated either in untransduced samples, or in eGFP-transduced and hCD18-transduced CD18^HYP^ Lin^−^ BM cells, indicating that neither the lentiviral transduction nor the hCD18 expression affected the clonogenic capacity of CD18^HYP^ hematopoietic progenitors (Supplementary Fig. S1C).

**Figure 4.**
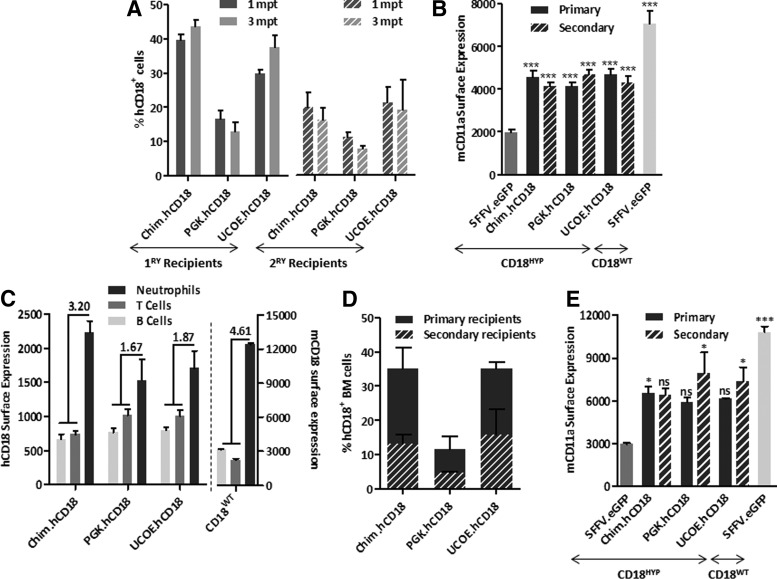
β_2_ integrins' expression in gene therapy (GT)-treated CD18^HYP^ mice. Lin^−^ cells from CD18^HYP^ mice were isolated and transduced with hCD18-LVs. Transduced cells were transplanted into lethally irradiated CD18^HYP^ mice. As control groups, CD18^HYP^ mice were transplanted with LV:SFFV.eGFP-transduced CD18^HYP^ and CD18^WT^ lin^−^ cells. **(A)** Percentage of hCD18^+^/mCD11a^+^ peripheral blood lymphocytes (PBLs) at 1 and 3 mpt in *ex vivo* primary and secondary GT-treated CD18^HYP^ mice. **(B)** Mean of mCD11a surface expression levels (mean fluorescence intensity, MFI) in PBLs at the different time points after transplant of transduced cells in primary (black) and secondary (scratched black) CD18^HYP^ recipient mice and eGFP control animals (dark and light gray). Presented results correspond to six independent experiments. The significance of differences between groups is expressed as ****p < 0.0001 and is always referred to the CD18^HYP^ + LV:SFFV.eGFP group. **(C)** Left *Y* axis shows the percentage of neutrophils, B-cells, and T-cells expressing hCD18 in *ex vivo* GT-treated primary recipients. Right *Y* axis shows physiological mCD18 expression pattern. The upper numbers show the ratio of myeloid and lymphoid CD18 expression. **(D)** Percentage of hCD18^+^ cells in total BMC population of primary and secondary recipients at 4 and 9 months posttransplantation, respectively. **(E)** mCD11a surface expression levels observed in the BM of transplanted mice. The total number of transplanted animals on each transduction group for any of the hCD18 therapeutic vectors was as follows: in primary transplants, 34 Chim, 17 UCOE, and 27 PGK; in secondary transplants, 7 Chim, 5 UCOE, and 3 PGK. In the case of control animals, 24 were transplanted with CD18^HYP^-GFP-transduced cells and 22 with CD18^WT^-GFP-transduced cells. The significance of differences between groups is expressed as *p < 0.05 and **p < 0.01. See Supplementary Table S1 for list of antibodies used.

In subsequent experiments, Lin^−^ BM cells from CD18^HYP^ mice were transduced with hCD18-LVs and then transplanted into lethally irradiated CD18^HYP^ recipients. As control groups, CD18^HYP^ mice were transplanted with either CD18^WT^ or CD18^HYP^ Lin^−^ BM cells transduced with an eGFP-LV. In all instances, CD18^HYP^ mice transplanted with corrected CD18^HYP^ BM cells showed the presence of hCD18^+^ leukocytes in PB (Fig. 4A). As expected, hCD18 was co-expressed with any of the three mCD11 subunits (Supplementary Fig. S2). Moreover, the ectopic expression of hCD18 significantly increased the surface expression of mCD11a, which was modestly expressed in eGFP-transduced CD18^HYP^ cells (Fig. 4B).

As in PB cells from WT mice, the analysis of PB cells from GT-treated CD18^HYP^ mice at 3 months after transplantation showed higher membrane levels of expression of hCD18 in myeloid cells as compared with B or T lymphoid cells, regardless of the promoter used. However, the ratio of hCD18 expression in myeloid versus lymphoid cells was higher in the Chim.hCD18 group (3.2-fold) than in the other groups (1.67 and 1.87), and closer to the ratio observed in WT mice (4.61-fold) (Fig. 4C). In all instances, VCNs/cell in the range of 0.4–0.9 were detected in peripheral blood lymphocytes (PBLs) from transplanted recipients, indicating that differences observed among the different LVs were not associated to differences in the numbers of integrated copies (Supplementary Fig. S3A). Four months after transplantation, primary recipients were culled and BMCs were also analyzed for hCD18 and mCD11a expression. Percentages of hCD18^+^ cells ranged from 11% to 35%, similarly to data obtained in PB (Fig. 4D). Furthermore, also a similar mCD11a upregulation was observed in total BMCs from GT-treated mice (Fig. 4E).

**Figure 5.**
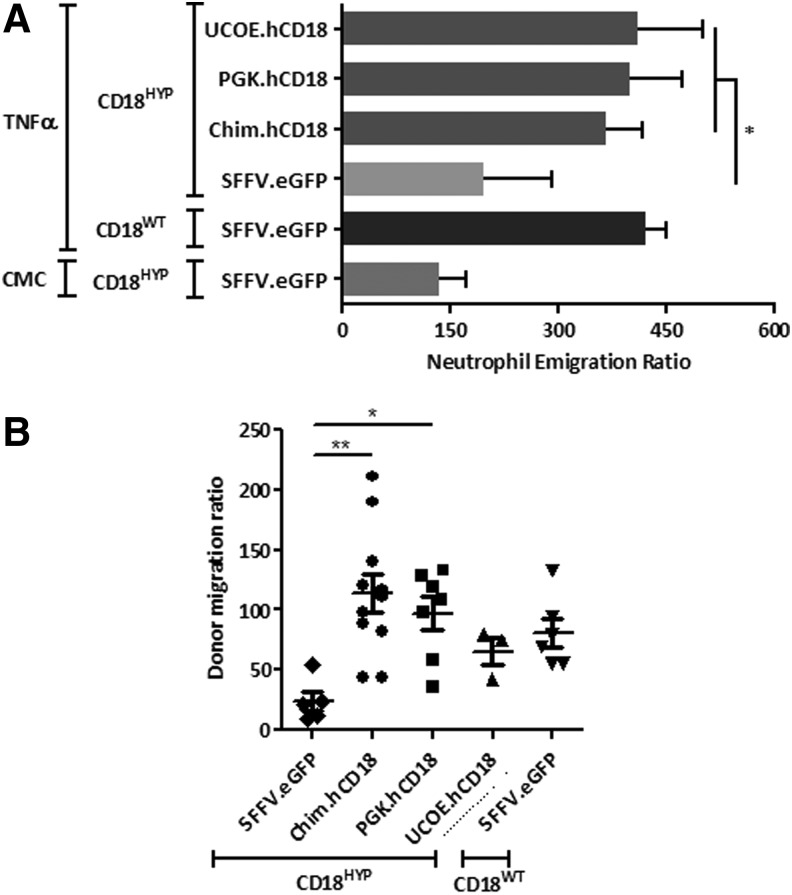
Correction of the LAD-I phenotype in *ex vivo* GT-treated CD18^HYP^ mice. **(A)** Air pouch-based inflammation model in *ex vivo* GT-treated primary CD18^HYP^ recipients and control mice treated either with TNFα (proinflammatory stimulus) or CMC (vehicle). Experiments were performed 4 months after transplantation. Neutrophil emigration ratio is calculated from the total cell number found within the pouch and the percentage of Gr1^+^ cells determined by flow cytometry. **(B)** Neutrophil migration to a lung inflammation model based on LPS intranasal administration. The donor neutrophil migration ratio is calculated from the percentage of Ly6G^+^ CD11c^−^ donor neutrophils found in the bronchoalveolar lavage and in peripheral blood. The significance of differences between groups is expressed as *p < 0.05 and **p < 0.01.

To evaluate if hCD18-LVs were able to transduce true LT-HSCs, total BMCs from primary recipients were re-transplanted into lethally irradiated CD18^HYP^ secondary recipients. As in primary recipients, a significant number of PB and BMCs from secondary recipients became hCD18^+^ (Fig. 4A) and upregulated mCD11a (Fig. 4B–E). Because CD18 deficiency leads to defects in neutrophil extravasation from PB to inflamed tissues, we investigated whether TNF-α-mediated neutrophil migration to a subcutaneous AP was restored in GT-treated CD18^HYP^ mice. A significant increment in the emigration ratio of neutrophils was observed in WT mice treated with TNFα, whereas no differences in this emigration ratio were observed in CD18^HYP^ mice (Fig. 5A). Significantly, when the same study was performed with GT-treated CD18^HYP^ mice, a significant increase in the TNF-α-induced emigration ratio was observed in all groups of GT-treated mice as compared with the eGFP-LV group (Fig. 5A). This indicates that ectopic expression of hCD18 was able to restore neutrophil migration of CD18^HYP^ mice.

We also conducted a second functional assay to evaluate whether the GT of CD18^HYP^ mice improved neutrophil migration ability based on an LPS-induced asthma model. Mice were intranasally treated with LPS, and a bronchoalveolar lavage (BAL) was performed to collect alveolar neutrophils that had migrated under the inflammatory stimulus. Whereas the donor migration ratio of CD18^HYP^ neutrophils was highly reduced in comparison with CD18^WT^ mice, a significant increment in this neutrophil migration ratio was observed in CD18^HYP^ mice treated with Chim and PGK hCD18-LVs (Fig. 5B).

### *In vitro* functional correction of LAD-I-like human CD34-derived granulocytes

**Figure 6.**
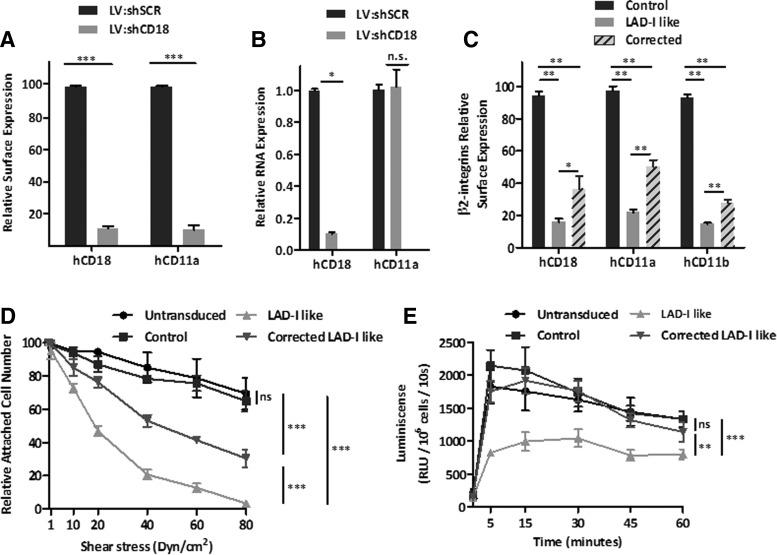
Generation of LAD-I-like hematopoietic cells. Expression of β_2_-integrin subunits in LV:shSCR and LV:shCD18-transduced healthy cord blood CD34^+^ after 6 days of culture. All transductions were performed at an MOI of 100 i.u./cell. **(A)** hCD18 and hCD11a surface expression levels. MFI values were normalized to MFI values of untransduced cells. **(B)** *hCD18* and *hCD11a* relative gene expression assessed by Q-PCR data using a ΔΔC_t_ method with GAPDH as an endogenous reference gene and normalized to untransduced cells. **(C)** β_2_-integrins expression in LV:shSCR control, LAD-I-like, and LV:Chim.hCD18-corrected neutrophils obtained by *in vitro* differentiation of cord blood CD34^+^ cells. Differentiation was carried out in the presence of SCF, IL3, and GCSF for up to 12 days. Expression levels were determined by flow cytometry as MFI and then normalized to the MFI of untransduced cells. **(D)** Flow chamber experiments with LV:shSCR control, LAD-I like, and LV:Chim.hCD18-corrected LAD-I-like neutrophils. Cells were allowed to attach to fibrinogen (ligand for CD11_b_:CD18 β2 integrins) and then nonadherent cells were removed at low flow. Subsequently, flow rates were increased in 10–20 dyn/cm^2^ increments in 1 min intervals to a maximum sheer stress of 80 dyn/cm^2^. The numbers of attached cells after each increment of shear stress were calculated as the percentage of total number of adherent cells in the same field after a low flow wash. **(E)** Respiratory burst on *in vitro* CD34^+^ cell-derived neutrophils. Respiratory burst response was induced by C3bi-opsonized zymosan (specifically recognized in a CD11/CD18-dependent manner) and detected by luminol-enhanced chemoluminescence. Luminescence detected for each sample was extrapolated for 10^6^ cells. The significance of differences between groups is expressed as *p < 0.05, **p < 0.01, and ***p < 0.001. See Supplementary Table S1 for list of antibodies used.

We next investigated whether hCD18-LVs restored the functional properties of LAD-like neutrophils. HD cord blood CD34^+^ cells were transduced with an LV expressing an shRNA against the CD18 mRNA (LV:shCD18). As a control, an LV carrying a scrambled shRNA (LV:shSCR) was also used. The surface expression of hCD18 and hCD11a was significantly reduced in LV:shCD18-transduced cells in comparison with LV:shSCR-transduced cells (Fig. 6A and Supplementary Methods). Moreover, when the expression of these β_2_-integrin subunits was analyzed by Q-PCR, a marked reduction in hCD18 expression was observed, whereas CD11a expression levels remained unchanged after LV:shCD18 transduction (Fig. 6B), consistent with the specificity of the shRNA against hCD18 mRNA.

To generate CD18-deficient neutrophils *in vitro*, CD34^+^ cells were cultured for 12 days in differentiation media. An aliquot of LV:shCD18-transduced cells was re-transduced with the therapeutic Chim.hCD18-LV before differentiation. Whereas the expression of hCD18, hCD11a, and hCD11b in LV:shSCR-transduced cells was significantly reduced compared with their control group (Fig. 6C), LAD-I-like cells transduced with the Chim.hCD18-LV showed a 43% recovery of control hCD18 levels. Similar increments in hCD11a and hCD11b expression were observed in corrected LAD-I-like neutrophils (Fig. 6C).

Finally, two different functional assays were carried out to evaluate if transduction of LAD-I-like CD34^+^ cells with the Chim.hCD18-LV had an impact on the functionality of *in vitro* differentiated neutrophils. First, the adhesion capacity in a dynamic setting was evaluated. In a flow chamber assay, differentiated cells were allowed to attach to fibrinogen under a fluid shear stress. LAD-I-like neutrophils showed a decreased ability to resist shear stress in comparison with control neutrophils (Fig. 6D). Such defects were significantly corrected in Chim.hCD18-LV-transduced LAD-I-like neutrophils, indicating that the ectopic expression of hCD18 restored, at least in part, the adhesion properties of LAD-I-like human neutrophils (Fig. 6D). In a second functional assay we evaluated the ability of *in vitro* differentiated neutrophils to undergo a respiratory burst. While control neutrophils were able to mount a rapid respiratory burst in response to opsonized zymosan, the respiratory burst of LAD-I-like neutrophils was clearly diminished. Interestingly, gene-corrected LAD-I-like neutrophils developed a respiratory burst that was similar to that observed in control cells (Fig. 6E).

## Discussion

LAD-I is a primary immunodeficiency leading to the death of most severely affected patients within the first two years of life. Despite the fact that allogeneic HSCT from HLA-identical related donors has resulted in survival rates from 71% to 91% in LAD-I patients,^31–34^ the access to these donors, together with complications associated to the procedure, constitutes the major limitation for this therapy. Hematopoietic GT with improved vectors has proved to be a promising approach for several monogenic disorders and a powerful alternative to allogeneic HSCT. Patients from different diseases, such as SCID-X1, ADA-SCID, WAS, B-Thal, ALD, or MLD, have gained significant clinical benefits from this new approach,^35–37^ without any evidence of insertional oncogenesis using later-generation vectors.^38^

Although CD18 is expressed in all leucocytes and also in HSPCs, levels of expression are particularly high in differentiated myeloid cells. Therefore, we aimed to investigate the therapeutic efficacy of different hCD18-LVs with either ubiquitous or myeloid-specific promoters. All previous reported data related to the PGK promoter^19–21,39^ and the A2UCOE promoter^26,22,23^ demonstrate their attractive safety profile. To mimic the physiological expression of CD18, we have also used a chimeric promoter with a preferential expression in myeloid cells.^27^

When our hCD18-LVs were tested *in vitro* in a human LAD-I cell line,^9^ full recovery, not only of hCD18 but also of hCD11a subunit expression, was obtained. The expression levels achieved were similar to those found in HD cells and led to complete phenotypic correction, as seen by the restoration of the aggregation capacity and the ability to bind to sICAM-1. These results indicated that all tested hCD18-LVs were able to restore β_2_ integrin expression and to correct the characteristic phenotype in LCs derived from a LAD-I patient.

Interestingly, when increasing doses of LV were used for transduction, surface expression levels of both subunits were rapidly saturated from an MOI of 10 i.u./cell onward. This indicates that the endogenous expression of hCD11 will limit the maximum amount of hCD18 ectopically expressed in the membrane, which implies a relevant safety issue favoring the GT of LAD-I patients

Our data in the CD18^HYP^ mouse model are consistent with previous studies showing that hCD18 can be expressed in the membrane of murine cells coupled to its mCD11 counterpart, indicating that both human and murine CD18 subunits might function similarly in a mouse environment.^8,10,40^ Our data additionally show that neutrophils derived from transduced CD18^HYP^ lin^−^ cells are able to upregulate hCD18 expression upon PMA stimulation. Moreover, when methylcellulose-based colony-forming cell assays were performed, no differences in the number of CFUs were observed among untransduced, GFP-transduced, or hCD18-transduced cells, indicating that neither the LV transduction nor the ectopic expression of hCD18 mediates significant hematopoietic toxicity.

When BM-purified LSK cells were transduced *in vitro* with any of the hCD18-LVs, the level of *hCD18* transgene expression obtained in all cases was low, though significant in LSK progenitor cells, while much higher in myeloid differentiated cells (Fig. 3D), thus mimicking the physiological expression of mouse and human CD18. After transplantation of hCD18-transduced CD18^HYP^ BM cells into CD18^HYP^ mice, hCD18^+^ cells could be detected in the PB of all GT-treated animals at a similar proportion, in both primary and secondary recipients. Moreover, these mice showed an upregulation in mCD11a expression in comparison with mice transplanted with eGFP-LV-transduced CD18^HYP^ cells, leading to the recovery of 50% of the levels found in control mice transplanted with CD18^WT^ cells. This upregulation was maintained long-term after transplantation as it was also observed in secondary recipients. hCD18 was expressed in T-cells, B-cells, and neutrophils, irrespective of the promoter used. However, neutrophils always showed higher hCD18 levels than B- and T-cells, as happens with the physiological mCD18 expression in these cells. This was observed in all GT-treated mice, no matter the nature of the promoter used. However, the ratio between the myeloid and lymphoid hCD18 expression was higher and closer to the physiological ratio in the group corresponding to the LV:Chim.hCD18.

To evaluate the efficacy of the hCD18-LV-mediated GT in the correction of the LAD-I phenotype in CD18^HYP^ mice, we analyzed neutrophil migration capacity in two inflammatory settings: the TNFα-induced AP inflammation model and the LPS-induced pulmonary inflammation model. In both of them, *ex vivo* GT-treated animals showed similar neutrophil migration to WT controls, indicating the recovery of the migration capacity of the donor neutrophils. It is important to highlight that our data show, for the first time in a mouse model, that GT restores *in vivo* functional defects associated to CD18 deficiency.

To confirm the efficacy of the hCD18-LVs in primary human hematopoietic cells, *CD18*-knockdown CD34^+^ cells were generated as target cells. This strategy has been previously used to generate human models to study the efficacy of GT for other diseases.^41,42^ Transduction of HD cord blood human CD34^+^ cells with an LV construct expressing an shRNA against hCD18 resulted in a 90% reduction of hCD18 expression levels determined by Q-PCR and by FACS, with a concomitant reduction in the hCD11a surface expression levels. LAD-I-like neutrophils were generated *in vitro* showing surface expression levels of hCD18, hCD11a, and hCD11b drastically reduced in comparison with control neutrophils. Moreover, LAD-I-like neutrophils were unable to properly bind and they could not mount a proper respiratory burst upon opsonized zymosan activation. Interestingly, when LAD-I-like CD34^+^ cells were transduced with the chim.hCD18-LV and differentiated *in vitro*, a significant re-expression of hCD18, hCD11a, and hCD11b was noted in the cell membrane of differentiated neutrophils. Moreover, an improved capacity to bind to fibrinogen, as well as a full restoration of the ability to mount a respiratory burst in response to opsonized zymosan, was noted in these samples.

The preclinical results obtained in this study suggest that any of the LVs generated in this study may constitute good candidates for the *ex vivo* hematopoietic GT of LAD-I. While the PGK-LVs have already demonstrated their efficacy and safety in different clinical trials, the Chim.hCD18-LV conferred CD18 expression levels slightly closer to the physiological ones. Taken together, although HSCT is nowadays the only curative therapy for LAD-I, data presented in this study allow us to strongly propose that GT with LVs expressing hCD18 would constitute an attractive approach for LAD-I patients lacking a related HLA-identical donor.

## Supplementary Material

Supplemental data
