# Supplementary material for: Lentiviral Vector-Mediated Correction of a Mouse Model of Leukocyte Adhesion Deficiency Type I
Source: Hum Gene Ther. 2016 Sep 1;27(9):668–78. doi: 10.1089/hum.2016.016 (PMC5035374; doi:10.1089/hum.2016.016)
Supplement: Supplemental data [file Supp_Data.pdf]

Supplementary Methods

hCD18-LVs cloning

The plasmid containing the SIN lentiviral backbone pCCL.PGK.eGFP.Wpre* was kindly provided by Dr. Luigi Naldini (San Raffaele-Telethon Institute for Gene Therapy, Milan, Italy). The plasmids containing Chim and A2UCOE promoters, pCCL.Chim.GFP.Wpre*[1] and pHR′.A2UCOE.GFP,[2] were kindly provided by Dr. Adrian Thrasher and Dr. Giorgia Santilli (Institute of Child Health–University College of London, London, United Kingdom). The plasmid containing the hCD18 (HsCD00044736) was obtained from PlasmID.

A fragment containing the lentiviral backbone and the PGK promoter was obtained by digestion of pCCL.PGK.eGFP.Wpre* plasmid with *Bam*HI. The generated overhang ends were blunted using a T4 DNA polymerase and treated with Antarctic phosphatase. HsCD00044736 plasmid was digested with *Psi*I to generate a fragment containing the *hCD18*. Both fragments were ligated to obtain the pCCL.PGK.CD18.Wpre* plasmid.

Lentiviral backbone was obtained by digestion of pCCL.PGK.eGFP.Wpre* plasmid with *Hpa*I and *Bam*HI restriction enzymes. On the other hand, a fragment containing Chim promoter together with the cPPT sequence was obtained from the pCCL .Chim.GFP.Wpre* plasmid by digestion with the same restriction enzymes. Both fragments were ligated to obtain the intermediate plasmid pCCL .Chim.Wpre*. This plasmid was digested with *Pst*I, and treated with Antarctic phosphatase. HsCD00044736 plasmid was digested with *Psi*I to generate a fragment containing the *hCD18*. Both fragments were ligated to obtain the pCCL.Chim .CD18.Wpre* plasmid.

Lentiviral backbone was obtained by digestion of pCCL.PGK.eGFP.Wpre* plasmid with *Eco*RV and *Xma*I restriction enzymes. On the other hand, a fragment containing UCOE promoter was obtained from the pHR′.A2UCOE.GFP plasmid following three steps: digestion with *Eco*RI, generation of blunt ends with T4 DNA polymerase, and digestion with *Age*I. Both lentiviral backbone and promoter fragments were ligated to obtain the intermediate plasmid pCCL.A2UCOE.Wpre*. This plasmid was digested with *Eco*RV and treated with Antarctic phosphatase. HsCD00044736 plasmid was digested with *Psi*I to generate a fragment containing the *hCD18*. Both fragments were ligated to obtain the pCCL.A2UCOE.CD18.Wpre* plasmid.

Animals

C57BL/6J (B6) were obtained from the Jackson Laboratory and bred at the CIEMAT animal facility (registration number 28079-21 A). Mice were maintained under high standard conditions (high-efficiency particulate [HEPA]-filtered air, regulated temperature of 22°C, light–dark cycle of 12 hr, and food and ultraviolet-irradiated water *ad libitum*) and routinely screened for pathogens. B6.129S7-*Itgb2*^{tm1Bay}/J colony founders (CD18 hypomorphic mice, CD18^{HYP}) were also obtained from the Jackson Laboratory and handled under sterile conditions, and maintained in microisolators.

All experimental procedures were carried out according to Spanish and European regulations (Spanish RD 53/2013 and Law 6/2013 that translate and comply with the European Directive 2010/ 63/UE about the use and protection of vertebrate

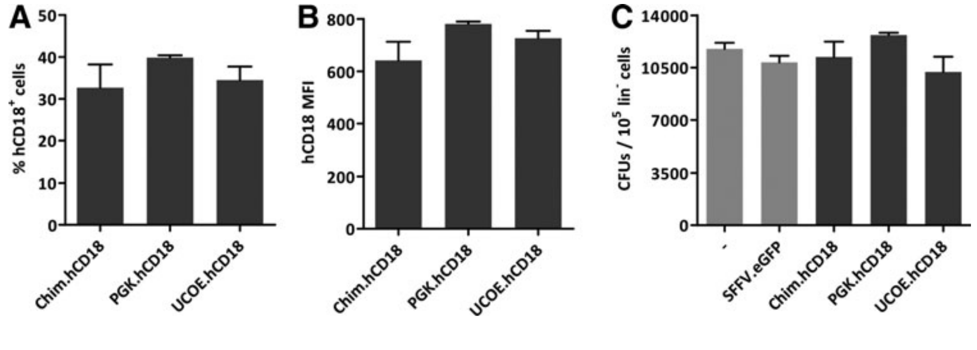

Supplementary Figure S1. *In vitro* transduction of mouse hematopoietic progenitors. **(A)** Percentage of hCD18^{+} CD18^{HYP} lin^{-} cells after myeloid differentiation. **(B)** mean fluorescence intensity (MFI) of hCD18^{+} CD18^{HYP} lin^{-} cells after myeloid differentiation. **(C)** Clonogenic assays in methylcellulose with transduced and untransduced CD18^{HYP} lin^{-} cells.

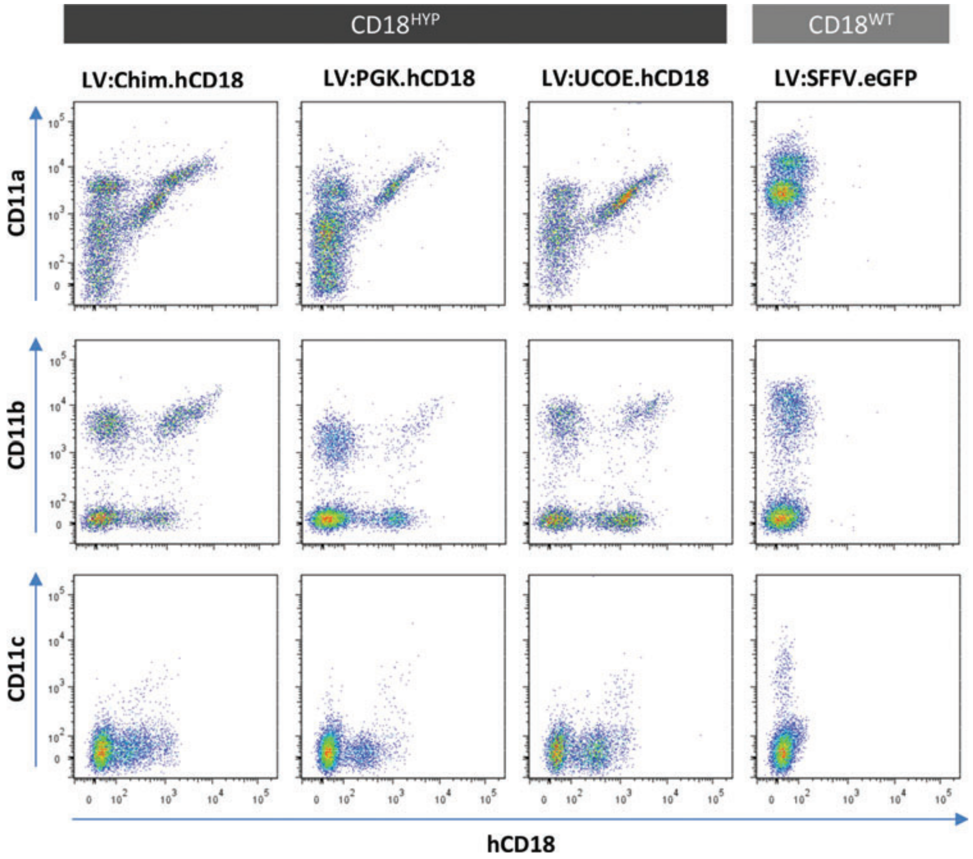

Supplementary Figure S2. β_2 integrins' expression of representative gene therapy (GT)-treated CD18^{HYP} primary recipients.

A

	LV:Chim.hCD18	LV:PGK.hCD18	LV:UCOE.hCD18
Primary recipients	0.897±0.430	0.738±0.162	0.438±0.005
Secondary recipients	0.457±0.145	0.358±0.112	0.350±0.087

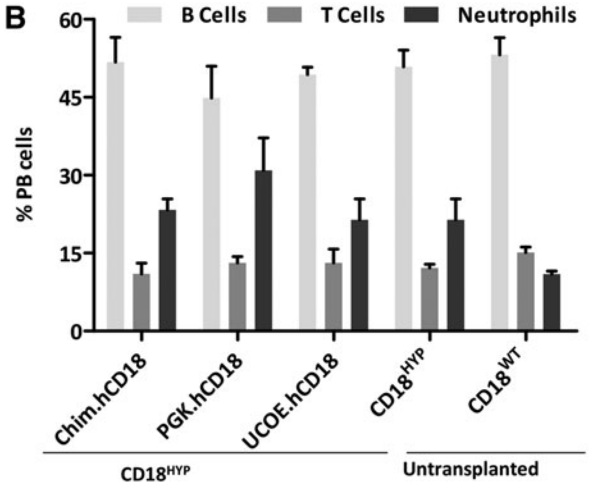

Supplementary Figure S3. Analyses of GT-treated CD18^{HYP} mice. **(A)** vector copy number (VCN) determination in gDNA extracted from peripheral blood lymphocytes (PBLs) from primary and secondary recipients at 3 months posttransplantation. **(B)** Multilineage distribution in peripheral blood (PB) at 4 months posttransplantation of secondary recipients and age-matched untransplanted CD18^{WT} and CD18^{HYP} mice.

Supplementary Table S1. *List of antibodies used*

	Reactivity	*Antigen*	*Conjugation*	*Vendor*	*Catalog No.*	*Clone*
Lin$^-$ sorting	Mouse	CD11b	FITC	BioLegend	101206	M1/70
	Mouse	CD45R/B220	FITC	Southern	1665-02	RA3-6B2
	Mouse	CD3	FITC	BD Pharmingen	553062	145-2C11
	Mouse	GR1(Ly6G)	FITC	BD Pharmingen	553127	RB6-8C5
	Mouse	Ter119	FITC	eBioscience	11-5921	Ter119
hCD18/hCD11a expression	Human	CD18	APC	BD Pharmingen	551060	6.7
in LCs	Human	CD11a	PE-Cy5	BD Pharmingen	551131	HI111
sICAM-1 binding assay	Human	Fc IgG	PE	eBioscience	12-4998-82	Polyclonal
Ex vivo GT experiments: hCD18	Human	CD18	APC	BD Pharmingen	551060	6.7
and mCD11 subunits	Human	CD18	FITC	BioLegend	302106	TS1.18
co-expression	Mouse	CD11a	PE-Cy7	BD Pharmingen	558191	2D7
	Mouse	CD11b	PE	BD Pharmingen	553311	M1/70
	Mouse	CD11c	APC	BD Pharmingen	550261	HL3
Ex vivo GT experiments: hCD18	Human	CD18	APC	BD Pharmingen	551060	6.7
expression in different	Mouse	CD3	PE	BD Pharmingen	553064	145-2C11
leukocyte subpopulations	Mouse	CD3	FITC	BD Pharmingen	553062	145-2C11
	Mouse	CD45R/B220	PE	BD Pharmingen	553090	RA3-6B2
	Mouse	GR1(Ly6G)	FITC	BD Pharmingen	553128	RB6-8C5
	Mouse	CD11b	FITC	BioLegend	101206	M1/70
Ex vivo GT experiments: hCD18	Human	CD18	APC	BD Pharmingen	551060	6.7
expression in BM LSK cells	Mouse	CD3	Biotin	BD Pharmingen	553060	145-2C11
	Mouse	Gr1(Ly6G)	Biotin	BD Pharmingen	553127	RB6-8C5
	Mouse	Mac1	Biotin	BD Pharmingen	553309	M1/70
	Mouse	CD45R/B220	Biotin	BD Pharmingen	553086	RA3-6B2
	Mouse	Ter119	Biotin	BD Pharmingen	553672	Ter119
	Mouse	CD117/cKit	PE	BD Pharmingen	553355	2B8
	Mouse	Sca1	FITC	BD Pharmingen	553335	E13-161.7

GT, gene therapy; LCs, lymphoblastic cells.

mammals used for experimentation and other scientific purposes).

Mouse Lin$^-$ cells

BM-derived hematopoietic Lin$^-$ cells were obtained from femora and tibiae using the moAbs listed on Supplementary Table S1 and FACS-sorted using a BD INFLUX (BD Biosciences).

Freshly isolated lin$^-$ cells were resuspended in StemSpan medium (StemCell Technologies) containing 100 ng/ml hIL-11 and 100 ng/ml mSCF (Eurobiosciences) and the lentiviral supernatant at 20–50 MOI. Lin$^-$ cells were allowed to grow in liquid culture for 7 days for flow cytometry analysis and vector copy number (VCN) determination.

For *in vitro* differentiation Lin$^-$ cells were cultured in neutrophil differentiation medium (RPMI supplemented with 20% HyClone, mIL-3 [10 ng/ml], hG-CSF [neulasta; 100 ng/ml], and 1% P/S [penicillin/streptomycin]).

For methyl-cellulose cultures 1.5×10^4 BMCs or 10^3 BM-derived lin$^-$ cells were seeded in methylcellulose-based medium with recombinant cytokines (Methocult M3534; StemCell Technologies). The number of CFUs was scored on day 7 under an inverted microscope.

Lymphoblastic cell lines

The lymphoblastoid cell line (LCs) from a LAD-I patient was kindly provided by Dr. Hickstein, and expresses no detectable CD18 mRNA or protein levels.[3] Healthy donor LCs were generated in Dr. Regueiro's laboratory from healthy samples and they were used as CD18^{WT} control in these experiments. All LCs were cultured in complete medium based on PRMI 1640 + GlutaMAX supplemented with 10% HyClone and 0.5% P/S. 2.5×10^5 cells were normally transduced O/N on RN (Takara Bio)-coated plates at the desired MOI.

PMA-induced aggregation

The aggregation assay was carried out as previously described.[4] Cells were washed two times and suspended in RPMI 1640 medium + GlutaMAX with 10% HyClone at a concentration of 2×10^6 cells/ml and added to a flat-bottomed 96-well plate. Then, 50 μl of PBS or 1 μg/ml PMA solution (Sigma-Aldrich) was added to each well and cells were incubated for 1 hr at 37°C. An inverted microscope was used to observe aggregation. In blocking studies, a mouse monoclonal antibody against hCD18 (LEAF purified antihuman CD18, clone TS1/18; BioLegend) was added at a final concentration of 7 μg/ml to the cell suspension 5 min before the addition of the PMA stimulus.

Soluble ICAM-1 binding assay

sICAM-1 binding assay was performed as formerly described.[5] sICAM-1 was prepared by incu-

bating 20 μg/ml recombinant human ICAM-1/Fc (Chimera; R&D System) with 100 μg/ml polyclonal PE-conjugated rabbit antihuman IgG Fc antibody (eBioscience) in 50 μl of RPMI 1640 medium + GlutaMAX containing 5% HyClone and HEPES 10 mM (Gibco/Life Technologies/Thermo Fisher Scientific) for 30 min at room temperature. An amount of 2×10^5 cells were resuspended in the sICAM mixture with or without β_2-activating agents (10 mM $MgCl_2$ and 3 mM EGTA) for 30 min at 37°C. Cells were washed with RPMI 1640 medium + GlutaMAX and analyzed by flow cytometry.

Purification of cord blood CD34$^+$ cells

Cord blood samples from healthy donors were obtained from the Madrid Community Transfusion Centre under their IRB approval and compelling with the Helsinki Declaration. Mononuclear cells were purified by Ficoll-Paque PLUS (GE Healthcare) density gradient centrifugation, and CD34$^+$ magnetic-labeled cells were selected using CD34 MicroBead Kit (MACS; Miltenyi Biotec). Purified CD34$^+$ cells were then evaluated for their purity by FACS. Purities from 80% to 95% were routinely obtained.

Transduction of human CD34$^+$ cells and neutrophil differentiation

Freshly isolated CD34$^+$ cells were cultured for 2 days in StemSpam supplemented with hSCF (300 ng/ml), hTPO (thrombopoietin, 100 ng/ml), hFlt3L (FMS-like tyrosine kinase 3 ligand, 100 ng/ml), and 1% P/S. At day 2 cells were collected, counted, and O/N transduced with shRNA-LVs at MOI of 100 on nontreated retronectin-coated 24-multiwell plates. The following day, cells were washed and resuspended in a fresh medium.

In the case of lentiviral correction, interfered-CD34$^+$ cells were allowed to grow for 3 additional days and then GFP$^+$ cells were FACS-sorted and resuspended in a fresh medium. The next 2 days cells were collected, counted, and transduced with CD18-LVs at MOI 100. The day after transduction, cells were washed and either resuspended in PBS for xenotransplant experiments or resuspended in neutrophil differentiation medium (IMDM supplemented with 20% Hyclone, hIL-3 [20 ng/ml], hSCF [20 ng/ml], hG-CSF [neulasta, 100 ng/ml], and 1% P/S) and allowed to proliferate for 12 days. These 12-day *in vitro*-differentiated neutrophils were used in two different functional assays: flow chamber assay and chemiluminescence assay.

Flow chamber assay

Flow chamber assays were performed as previously described.[6] An amount of 1×10^5 12-day differentiated neutrophils were mixed with 1×10^5 control untransduced neutrophils and resuspended in 500 μl of RPMI 1640 medium + GlutaMAX with 10% Hyclone. Plastic slides were coated with fibrinogen (2.5 mg/ml) for 1 hr at 37°C. Flow chambers (Department of Medical Engineering, Imperial College School of Medicine, London, United Kingdom) were assembled with 0.2 mm spacers, precoated slides, and an exit tubing (Medex/Smiths Medical). Four 50 ml syringes were filled with PBS and connected to the chamber using tubing (Medex/Smiths Medical), a 3-way tap (BD/Becton, Dickinson and Company), and Y-connectors (Alaris/CareFusion). The tubing and chambers were primed to eliminate bubbles and mounted on a microscope stage inside of an environmental chamber (37°C). Syringes were assembled on a BS-9000-6 multisyringe programmable syringe pump with flow-rate control (Braintree Scientific). Cells were injected through the three-way tap and passed into the chamber with about 1 ml PBS from the syringes. After 10 min adherence, PBS was pumped into the chamber at low flow (1 dyn/cm^2/min) for 1 min to remove nonadherent cells. Afterward, flow rates were augmented in 10–20 dyn/cm^2 increments in 1 min intervals to a maximum sheer stress of 80 dyn/cm^2. Images of bright field and eGFP fluorescence were acquired after each flow rate increment using a Zeiss Axiovert 135 microscope with a 10× phase contrast lens (Zeiss), a digital camera (Hamamatsu Photonics), and Volocity 4.2 software (PerkinElmer).

The shear stress for each flow rate was calculated from the following formula:

$$\text{Shear stress} = \frac{6\times\text{flow rate}\times\text{viscosity}}{\text{channel width}\times(\text{channel height})^2}$$

where shear stress is expressed in dyn/cm^2, flow rate is expressed in ml/sec, viscosity of PBS is 0.0076 poise, channel height = chamber width = 0.7 cm, and channel height = spacer height = 0.02 cm.

The number of cells detaching at each increment of shear stress was calculated and expressed as a percentage of the total number of adherent cells in the same field immediately after the low flow wash. eGFP$^+$ cells correspond to the LV:shSCR and LV:sh10-transduced cells and eGFP$^-$ cells correspond to the untransduced cells.

Chemiluminescence assay of neutrophil respiratory burst

Twelve-day *in vitro*-differentiated neutrophils were compared in a chemiluminescence assay of respiratory burst activity in response to complement-opsonized zymosan. Activation of the respiratory burst in response to C3bi-opsonized zymosan has been shown to be a CD11/CD18-mediated function

activity.[7,8] Luminol-enhanced chemiluminescence was used as a sensitive measure of the respiratory burst of human phagocytes as previously described.[9] An amount of 1×10^5 cells were preincubated for 15 min in a 160 μl of RPMI 1640 medium + GlutaMAX with 15 μg/ml human serum albumin at room temperature in an Isoplate-96 Microplate White Frame Clear Well (PerkinElmer). At the beginning of the assay, 10 μM luminol (Sigma-Aldrich) and 1 mg/ml opsonized zymosan were added to the reaction mixture. Luminol-enhanced chemiluminescence was read for 10 sec intervals at the designated time points with a Genios Pro reader (Tecan). The assay was performed at room temperature and chemiluminescence was reported as relative light units (RLU)/10^6 cells/10 sec.

Zymosan (Sigma-Aldrich) was opsonized with human serum as previously described.[10] Briefly, zymosan was resuspended in PBS at 20 mg/ml, heated and shaken at 100°C for 20 min, sonicated for 60 s, and washed in PBS by centrifugation (300 g, 2 min). Finally zymosan was resuspended in fresh PBS at 20 mg/ml and incubated with an equal volume of pooled human serum. The mixture was incubated at 37°C for 1 hr (keeping zymosan in suspension). Opsonized zymosan was washed twice in PBS by centrifugation (300 g, 2 min), resuspended in PBS at 10 mg/ml, and stored at –80°C.

Flow cytometry analyses

Flow cytometry analyses were performed in the LSRFortessa cell analyzer (BD/Becton, Dickinson and Company). Off-line analysis was performed with the FLowJo Software v7.6.5. (Tree Star).

A minimum of 10^4–10^5 viable cells were normally acquired. Samples were always resuspended in flow cytometry buffer (PBS containing 0.5% BSA and 0.05% sodium azide) containing 1 μg/ml DAPI as a viability marker. In the case of PB and BM samples, erythrocytes were lysed in ammonium chloride lysis solution (0.155 mM NH_4Cl, 0.01 mM $KHCO_3$, 10^{-4} mM EDTA) before antibody staining. Supplementary Table S1 summarizes all the labeled antibodies used in the different experiments. Fluorochrome-conjugated streptavidins were used as a second-step reagent to detect biotinylated primary antibodies.

Production of lentiviral vectors

All SIN LVs were generated by a second-generation packaging system in which 293T cells were transiently transfected with the transfer, helper (pCMVdR8.74), and envelope (pMD2.VSVg) plasmids, obtaining VSV-G-pseudotyped lentiviral particles. The pMD2.VSVg and pCMVdR8.74 plasmids used were obtained from PlasmidFactory. Transfections were performed after the $CaCl_2$ DNA precipitation method previously described.[11,12]

Viral titers were determined by infection of HT1080 cells with serial dilutions of the supernatants. Those vectors containing a GFP-expressing cassette were tittered by FACS and the rest tittered by Q-PCR.

REFERENCES

1. Santilli G, Almarza E, Brendel C, et al. Biochemical correction of X-CGD by a novel chimeric promoter regulating high levels of transgene expression in myeloid cells. Mol Ther 2011;19:122–132.
2. Zhang F, Thornhill SI, Howe SJ, et al. Lentiviral vectors containing an enhancer-less ubiquitously acting chromatin opening element (UCOE) provide highly reproducible and stable transgene expression in hematopoietic cells. Blood 2007;110:1448–1457.
3. Kishimoto TK, Hollander N, Roberts TM, et al. Heterogeneous mutations in the beta subunit common to the LFA-1, Mac-1, and p150,95 glycoproteins cause leukocyte adhesion deficiency. Cell 1987;50:193–202.
4. Rothlein R, Springer TA. The requirement for lymphocyte function-associated antigen 1 in homotypic leukocyte adhesion stimulated by phorbol ester. J Exp Med 1986;163:1132–1149.
5. Tang RH, Tng E, Law SK, et al. Epitope mapping of monoclonal antibody to integrin alphaL beta2 hybrid domain suggests different requirements of affinity states for intercellular adhesion molecules (ICAM)-1 and ICAM-3 binding. J Biol Chem 2005;280:29208–29216.
6. Burns S, Hardy SJ, Buddle J, et al. Maturation of DC is associated with changes in motile characteristics and adherence. Cell Motil Cytoskeleton 2004;57:118–132.
7. Arnaout MA, Todd RF 3rd, Dana N, et al. Inhibition of phagocytosis of complement C3- or immunoglobulin G-coated particles and of C3bi binding by monoclonal antibodies to a monocyte-granulocyte membrane glycoprotein (Mol). J Clin Invest 1983;72:171–179.
8. Hickstein DD, Locksley RM, Beatty PG, et al. Monoclonal antibodies binding to the human neutrophil C3bi receptor have disparate functional effects. Blood 1986;67:1054–1062.
9. Bauer TR, Schwartz BR, Liles WC, et al. Retroviral-mediated gene transfer of the leukocyte integrin CD18 into peripheral blood CD34+ cells derived from a patient with leukocyte adhesion deficiency type 1. Blood 1998;91:1520–1526.
10. Tlili A, Erard M, Faure MC, et al. Stable accumulation of p67phox at the phagosomal membrane and ROS production within the phagosome. J Leukoc Biol 2012;91:83–95.
11. Dull T, Zufferey R, Kelly M, et al. A third-generation lentivirus vector with a conditional packaging system. J Virol 1998;72:8463–8471.
12. Vigna E, Amendola M, Benedicenti F, et al. Efficient Tet-dependent expression of human factor IX *in vivo* by a new self-regulating lentiviral vector. Mol Ther 2005;11:763–775.
